# The role of peripheral blood eosinophil counts in acute Stanford type A aortic dissection patients

**DOI:** 10.3389/fsurg.2022.969995

**Published:** 2022-08-30

**Authors:** Xichun Qin, Yaxuan Gao, Yi Jiang, Feng Zhu, Wei Xie, Xinlong Tang, Yunxing Xue, Dongjin Wang, Hailong Cao

**Affiliations:** ^1^ Department of Cardio-Thoracic Surgery, Nanjing Drum Tower Hospital, The Affiliated Hospital of Nanjing University Medical School, Nanjing, China; ^2^ Institute of Cardiothoracic Vascular Disease, Nanjing University, Nanjing, China; ^3^ Department of Cardio-Thoracic Surgery, Nanjing Drum Tower Hospital Clinical College of Nanjing Medical University, Nanjing, China

**Keywords:** acute Stanford-A aortic dissection, eosinophil, mortality risk, blood coagulation, thrombosis

## Abstract

**Background:**

Acute Stanford-A aortic dissection (AAAD) is a devastating cardiovascular condition with high mortality, therefore identifying risk prognosis factors is vital for the risk stratification of patients with AAAD. Here, we investigated peripheral blood eosinophil (EOS) counts in patients with AAAD and their possible biological implications.

**Methods:**

We performed a single center retrospective cohort study. From 2011 to 2021, a total of 1,190 patients underwent AAAD surgery. Patients were categorized first by death and then admission EOS counts (0.00 × 10^9^/L or >0.00 × 10^9^/L). Demographics, laboratory data, and outcomes were analyzed using standard statistical analyses. Ascending aorta specimens were used for western blotting and histological assessments.

**Results:**

Death group patients had lower EOS counts than the non-death group (*P* = 0.008). When patients were stratified using mean blood EOS counts: 681 patients had low (0.00 × 10^9^/L) and 499 had high (>0.00 × 10^9^/L) counts. Patients with low EOS counts at admission were more likely to have a higher mortality risk (*P* = 0.017) and longer treatment in the intensive care unit (ICU) days (*P *= 0.033) than patients with normal EOS counts. Also, the five blood coagulation items between both groups showed significantly different (*P* < 0.001). Hematoxylin & eosin-stained cross-sections of the ascending aorta false lumen showed that EOSs were readily observed in thrombi in the false lumen of the aorta.

**Conclusions:**

Peripheral blood EOS counts may be involved in thrombosis and could be an effective and efficient indicator for the diagnosis, evaluation, and prognosis monitoring of patients with AAAD.

## Introduction

Acute aortic dissection (AAD) is a serious life-threatening disease, with a gradually increasing incidence rate in recent years (1). Depending on the rupture site, AAD is classified as Stanford-A when it involves the thoracic segment of the ascending aorta and/or the aortic arch, and Stanford-B when it involves the descending aorta and/or the thoracoabdominal aorta (2). Acute Stanford-A aortic dissection (AAAD) is the most common type, accounting for 75% of all cases. If treatment is not timely, many patients with AAAD will die suddenly due to aortic wall rupture. The mortality rate is as high as 90% (3). With the establishment of regional referral centers, more patients with AAAD can now receive timely surgical intervention, but all-cause mortality remains up to 10%–30% (4, 5).

Patients with AAAD usually undergo vital sign monitoring and blood tests upon admission in the emergency department, therefore, more clinicians are now heeding circulating biomarkers, such as routine bloods, in an attempt to analyze or predict the risk of death in these patients (6, 7). Laboratory test results are easily generated and do not impose additional risk and financial burden on patients. Many clinical and basic research studies have reported that inflammation has important roles in cardiovascular disease, with several inflammatory factors predicting cardiovascular disease (CVD) progression and prognosis (8, 9). These findings are also applicable to AAAD (10). White blood cells (WBC) are generally elevated in patients with AAAD and associated with a more severe prognosis and higher mortality (11). Eosinophils (EOS) are a type of WBC, and are implicated in several CVDs. For example, high blood EOS counts are positively associated with major cardiovascular risk factors and CVD prevalence (12), low EOS counts are negatively associated with peripheral arterial disease (13), and higher blood EOS counts are recorded in patients with abdominal aortic aneurysm when compared with normal controls, and can act as independent risk factors for the condition (14). However, we found that in the majority of patients with AAAD, EOS counts were <0.02 × 10^9^/L, and even dropped below the lower limit of detection (denoted as 0.00 × 10^9^/L). Compared to the change in the WBC counts, the EOS counts which were “0.00” seemed to be more striking.

While research on the pathogenesis of AAAD has progressed, EOS alterations during the condition and their possible biological ramifications are rarely considered. In this study, we investigated this phenomenon, characterized EOS changes of AAAD patients, and examined their roles.

## Methods

Materials and extension methods are described in Supplementary Materials.

### Study design

The principal study was a single-center, retrospective study. That study was approved by the Ethics Committee of the Affiliated Drum Tower Hospital of Nanjing University Medical School.

### Study patients

From January 2011 to December 2021, 1,190 AAAD surgeries were performed in Nanjing Drum Tower Hospital. An AAAD diagnosis was confirmed by aorta angiography using multi-detector computed tomographic scanning. Stanford type A (DeBakey I and II) dissection involved the ascending aorta and/or the aortic arch according to previously published criteria. All patients underwent serological testing in the emergency department preoperatively, and the results were considered as admission laboratory data. Patients were excluded if they died preoperatively or had no laboratory data (routine blood examinations including EOS counts) or had taken medications such as aspirin, antibiotics, and glucocorticoids affecting blood counts. The study was reviewed by the hospital Ethics Committee. Laboratory analytes measured at admission included routine blood examinations, biochemical analysis, D-dimer, cTNT, and five blood coagulation items.

### Clinical character

Patient demographic data, including age, gender, body mass index (BMI), disease onset, and previous medical history, including hypertension, diabetes, atrial fibrillation, Marfan syndrome, chronic obstructive pulmonary disease, coronary artery bypass grafting history, and smoking and drinking status. Other clinical characteristics included symptoms and signs at admission. The rationale for surgery and the surgical strategy were both determined by attending surgeons.

### Primary and secondary outcomes

The primary study outcome was inpatient mortality which was defined as all-cause death. Secondary outcomes included the time in the intensive care unit (ICU), post- tracheostomy, post-stroke, and post-intracranial hemorrhage after surgeries during the index hospitalization.

### Statistical analysis

Continuous variables were expressed as the mean ± standard deviation and compared using t tests if they were normally distributed. Skewed continuous variables were analyzed as the median and inter quartile range (IQR), and comparisons were made using the Mann-Whitney U test, with significance accepted at *P *< 0.05. Binary and categorical variables were expressed as counts and percentages, and compared using the *χ*^2^ or Fisher’s exact test. A two-sided *α* < 0.05 was considered statistically significant. Statistical analyses were performed in SPSS version 26.0 (SPSS, Inc., Chicago, IL, United States).

## Results

In total, 1,190 patients with AAAD were investigated. We excluded patients with no laboratory data and who had taken medications affecting blood counts. Finally, 1,180 patients with AAAD were analyzed, of whom 1,009 survived and 171 died. Next, we divided AAAD patients into death and non-death groups. Demographics were similar between groups, except for age (*P* < 0.001) and hypertension history (*P* = 0.005). Moreover, patients in the death group were more likely to have hypotension (22 [12.2%] vs. 66 [6.5%], *P* < 0.01) and preoperative limb ischemia before surgery (44 [24.4%] vs. 135 [13.4%], *P* = 0.001) (Table 1).

**Table 1:** Demographics and characteristics of non-death and death AAD patient.

Variables	Total (*n* = 1180)	Death (*n* = 171)	Non-death (*n* = 1009)	*P* value
Age, years	53 (44–63)	58 (49–68)	52 (44–62)	<0.001*
Gender (male/female)	883/297	124/47	759/250	0.505
BMI (kg/m^2^)	25.3 (23.05–28.02)	25.39 (23.24–28.22)	25.40 (23.03–27.99)	0.811
Smoking history	293 (24.8%)	43 (25.1%)	250 (24.8%)	0.924
Drinking history	215 (18.2%)	36 (21.1%)	179 (17.7%)	0.335
**Complications**				
HTN	878 (74.4%)	138 (80.7%)	740 (73.3%)	0.046*
DB	41 (3.4%)	2 (1.2%)	39 (3.9%)	0.075
COPD	10 (0.8%)	2 (1.2%)	8 (0.8%)	0.645
AF	12 (1.0%)	3 (1.8%)	9 (0.9%)	0.398
Marfan	23 (1.9%)	1 (0.6%)	22 (2.2%)	0.234
CABG history	1 (0.1%)	1 (0.6%)	0 (0.0%)	0.145
Stroke history	35 (3.0%)	6 (3.5%)	29 (2.9%)	0.808
**Symptoms**				
Pain	1,101 (93.3%)	164 (95.9%)	937 (92.9%)	0.184
Chest pain	1,011 (85.7%)	147 (86.0%)	864 (85.6%)	1.000
Back pain	536 (45.4%)	77 (45.0%)	459 (45.5%)	0.934
Abdominal pain	79 (6.7%)	15 (8.8%)	64 (6.3%)	0.247
Nausea	175 (18.1%)	25 (18.4%)	150 (18.1%)	1.000
Vomiting	146 (15.1%)	20 (14.7%)	126 (15.1%)	0.200
Stroke hemiplegia	17 (1.4%)	6 (3.5%)	11 (1.1%)	0.026*
**Sign**				
Hypotension	88 (7.5%)	22 (12.9%)	66 (6.5%)	0.005*
Heart rate	80 (69–94)	84 (70–99)	80 (69–93)	0.127
Pericardial effusion	809 (68.6%)	116 (67.8%)	693 (68.7%)	0.859
Preoperative limb ischemia	174 (14.5%)	40 (23.4%)	134 (13.3%)	0.001*

BMI, body mass index; HTN, hypertension; DB, diabetes; COPD, chronic obstructive pulmonary disease; AF, atrial fibrillation; CABG, coronary artery bypass grafting.

* Statistically significant values.

In terms of laboratory data, normal EOS counts were between 0.02–0.52 × 10^9^/L, however, over 80% of EOS counts below 0.02 × 10^9^/L, and 57.7% of AAAD patients dropped below the lower limit of detection (0.00 × 10^9^/L). Moreover, when EOS counts were considered as a continuous variable, we identified significant differences between death and non-death patients (*P* = 0.008), and EOS percentages lower in the death group (*P* = 0.002). When EOS counts were considered a categorical variable, we identified differences between groups (*P* = 0.038/0.017). Higher WBC and neutrophil counts were identified in the death group. In contrast, lymphocyte and monocyte counts in both groups were not significantly different (Table 2). These data suggested that reduced EOS counts were almost a universal feature in patients with AAAD.

**Table 2:** Laboratory test results of non-death and death AAD patients.

Variables	Total (*n* = 1180)	Death (*n* = 171)	Non-death (*n* = 1009)	*P* value
WBC (4.0–10.0 × 10^9^/L)				
<4	14 (1.2%)	6 (3.5%)	8 (0.8%)	<0.001*
4–10	451 (38.2%)	48 (28.1%)	403 (39.9%)	
>10	715 (60.6%)	117 (68.4%)	598 (59.3%)	
Neutrophils (2.0–6.0 × 10^9^/L)				
<2	4 (0.3%)	3 (1.8%)	1 (0.1%)	0.012*
2–6	175 (14.8%)	22 (12.9%)	153 (15.2%)	
>6	1,001 (84.8%)	146 (85.4%)	855 (84.7%)	
Lymphocytes (1.0–3.5 × 10^9^/L)				
<1	735 (62.3%)	105 (61.4%)	630 (62.4%)	0.796
≥1	445 (37.7%)	66 (38.6%)	379 (37.6%)	
Monocytes (0.1–0.6 × 10^9^/L)				
<0.6	500 (42.4%)	68 (39.8%)	432 (42.8%)	0.456
≥0.6	680 (57.6%)	103 (60.2%)	577 (57.2%)	
Eosinophils (0.02–0.52 × 10^9^/L)				
0	681 (57.7%)	113 (66.1%)	568 (56.3%)	0.038*
0–0.02	276 (23.4%)	38 (22.2%)	238 (23.6.%)	
0.02–0.52	221 (18.7%)	20 (11.7%)	201 (19.9%)	
>0.52	2 (0.2%)	0 (0.0%)	2 (0.2%)	
Eosinophils (0.02–0.5 × 10^9^/L)				
0	681 (57.7%)	113 (66.1%)	568 (56.3%)	0.017*
>0	499 (42.3%)	58 (33.9%)	441 (43.7%)	
Eosinophils (continuous)	0.00 (0.00–0.02)	0.00 (0.00–0.01)	0.00 (0.00–0.02)	0.008*
WBC (continuous)	11.2 (8.6–14.1)	12.4 (8.7–15.4)	11 (8.5–13.8)	0.002*
Neutrophils (continuous)	9.7 (7.13–12.4)	10.8 (7.2–13.9)	9.5 (7.1–12.2)	0.002*
Lymphocytes (continuous)	0.8 (0.5–1.1)	0.8 (0.5–1.1)	0.8 (0.5–1.1)	0.155
Monocytes (continuous)	0.6 (0.4–0.9)	0.4 (0.7–1.0)	0.6 (0.4–0.9)	0.741
Hemoglobin (115–150 g/L)	122 (101–139)	120 (93–139)	154 (101.5–139)	0.099
Platelet (100–400 × 10^9^/L)	137 (98.25–177)	120 (72–172)	139 (102–180)	<0.001*
Neutrophils (40%–70%)	87.20 (81.83–90.30)	87.80 (83.30–89.90)	87.00 (81.70–90.40)	0.355
Lymphocytes (20%–50%)	6.95 (4.80–10.40)	6.50 (4.60–9.90)	7.00 (4.90–10.40)	0.241
Monocytes (3%–10%)	5.7 (4.1–7.6)	5.6 (4.3–7.2)	5.7 (4.1–7.8)	0.284
Eosinophils (0.4%–8%)	0.00 (0.00–0.10)	0.00 (0.00–0.10)	0.00 (0.00–0.20)	0.002*
C-reactive protein (0–10 mg/L)	45.55 (6.68–108.35)	53.75 (6.68–117.3)	45.15 (6.63–106.45)	0.619
Albumin (30–55 g/L)	37.00 (33.50–19.90)	34.95 (30.43–38.88)	37.30 (33.80–40.10)	<0.001*
D-dimer (0–0.5 mg/L)	5.19 (2.82–11.61)	8.26 (4.29–21.08)	4.80 (2.60–9.86)	<0.001*
CTNT (0.02–0.13 ug/L)	0.028 (0.01–0.16)	0.069 (0.019–0.35)	0.025 (0.01–0.131)	<0.001*
PT (10–15 s)	12.6 (11.7–14.2)	13.7 (12.5–16.6)	12.5 (11.6–14.0)	<0.001*
INR (0.8–1.3)	1.11 (1.02–1.24)	1.20 (1.09–1.46)	1.09 (1.01–1.22)	<0.001*

CTNT, troponin T; PT, prothrombin time; INR, international normalized ratio; WBC, white blood cell.

* Statistically significant values.

We selected tissue from patients with AAAD (EOS count = 0.00 × 10^9^/L) and normal human ascending aorta tissue (EOS count = 0.02–0.52 × 10^9^/L), and investigated Siglec-8 expression (EOS marker protein). Interestingly, in ascending aortic tissue from patients with AAAD, we observed no abnormal Siglec-8 expression (Supplementary Figures S1A,B). Additionally, we observed no EOS infiltration or enrichment in AAAD patient’s aorta tissue using H&E staining (Supplementary Figure S1C).

To further investigate the role of EOS counts in AAAD, we divided patients into two groups based on circulating EOS counts at admission; a lower EOS group (0.00 × 10^9^/L) and a higher EOS group (>0.00 × 10^9^/L). No significant clinical differences were identified between groups. Similarly, no differences in age (*P* = 0.628), BMI (*P* = 0.226), and the median interval from onset to hospitalization (*P* = 0.196) were identified.

The proportion of patients in the lower EOS group, who developed pericardial effusion (65.8% vs. 72.3%, *P* = 0.017) was significantly lower than the higher EOS group. Moreover, patients in the lower EOS group who developed cerebral ischemia attack (10.0% vs. 5.8%, *P* = 0.010) were higher than the higher EOS group (Table 3).

**Table 3:** The effects of different eosinophil levels on the clinical characteristics, primary and secondary outcomes of patients with AAD.

Variables	Total (*n* = 1180)	EOS of 0.00 (*n* = 681)	EOS > 0.00 (*n* = 499)	*P* value
Age, years	53 (44–63)	53 (45–63)	53 (44–63)	0.628
Gender (male/female)	883/297	483/198	400/99	<0.001*
BMI (kg/m^2^)	25.39 (23.05–28.02)	25.35 (22.99–27.91)	25.72 (23.18–28.34)	0.226
From onset to admission	10 (6–18)	10 (7–15)	9 (6–22)	0.196
Leg pain				
Left	42 (3.6%)	26 (3.8%)	16 (3.2%)	0.032*
Right	19 (1.6%)	6 (0.9%)	13 (2.6%)	0.010*
Both	10 (0.8%)	3 (0.4%)	7 (1.4%)	
Cerebral ischemia attack	97 (8.2%)	68 (10.0%)	29 (5.8%)	
Hypotension	88 (7.5%)	57 (8.4%)	31 (6.2%)	0.163
Pericardial effusion	809 (68.6%)	448 (65.8%)	361 (72.3%)	0.017*
Mortality	171 (14.5%)	113 (16.6%)	58 (11.6%)	0.017*
ICU admission	5 (3–8)	5 (3–8)	4 (3–7)	0.033*
Post tracheostomy	62 (5.4%)	42 (6.3%)	20 (4.1%)	0.091
Post stroke	79 (6.7%)	44 (6.5%)	35 (7.1%)	0.841
Post intracranial hemorrhage	13 (1.1%)	8 (1.2%)	5 (1.0%)	1.000

BMI, body mass index; ICU, intensive care unit.

* Statistically significant values.

By examining primary and secondary outcomes in both EOS groups, patients with an admission EOS count of 0.00 × 10^9^/L had higher mortality rates (113 [16.6%] vs. 58 [11.6%], *P* = 0.017) and increased ICU treatment days [5.0 days, (IQR 3.0–8.0) vs. 4.0 days, (IQR 3.0–7.0), *P* = 0.033]. No significant differences in tracheotomy (42 [6.3%] vs. 20 [4.1%], *P* = 0.091) and intracranial hemorrhage rates (8 [1.2%] vs. 5 [1.0%], *P* = 1.000) were identified (Table 3).

The results of univariate regression analysis of predictors of mortality were shown in Table 4. Admission EOS count was associated with mortality both as a continuous variable (OR = 0.004, 95% CI 0.000–0.241, *P* = 0.006) and as a cutoff value of >0.00 × 10^9^/L (OR = 0.661, 95% CI 0.470–0.929, *P* = 0.017). Other risk factors associated with all-cause mortality included age, hypertension and WBC count. Add risk factors which *P**** ***< 0.05 into multivariable logistic regression models. Multivariable-adjusted ORs for mortality according to per 1.0 × 10^9^ cells/L increase, the cutoff value of 0.00 × 10^9^/L, were presented in Table 5. Admission EOS count was an independent predictor of death when considered as a continuous variable (OR = 0.010, 95% CI 0.000–0.650, *P *= 0.031) or as a categorical variable (OR = 1.46, 95% CI 1.033–2.070, *P *= 0.032) using the cutoff value of 0.00 × 10^9^/L after adjustment for age, hypertension and WBC count.

**Table 4:** Predictors of mortality after AAAD surgery in univariate logistic regression.

Variables	Odds Ratio	95%CI	*P* value
Age, years	1.036	1.023–1.049	<0.001*
Gender, (male/female)	0.869	0.603–1.252	0.451
BMI (kg/m^2^)	1.006	0.967–1.046	0.775
Hypertension	1.520	1.014–2.278	0.041*
WBC (continuous)	1.060	1.021–1.100	0.002*
Eosinophils (continuous)	0.004	0.000–0.241	0.006*
Eosinophils =0 cells/L	Reference		
Eosinophils >0 cells/L	0.661	0.470–0.929	0.017*
Neutrophils (continuous)	1.000	0.994–1.006	0.986

* Statistically significant values.

**Table 5:** Predictors of mortality after AAAD surgery in multivariable logistic regression.

Variables	Odds Ratio	95%CI	*P* value
**Model 1**			
Age	1.040	1.027–1.054	<0.001*
Hypertension	1.373	0.907–2.077	0.134
WBC (continuous)	1.075	1.033–1.119	<0.001*
Eosinophils (continuous)	0.010	0.000–0.650	0.031*
**Model 2**			
Age	1.04	1.027–1.055	<0.001*
Hypertension	1.37	0.906–2.075	0.135
WBC (continuous)	1.08	1.039–1.124	<0.001*
Eosinophils =0 cells/L	Reference		
Eosinophils >0 cells/L	1.46	1.033–2.070	0.032*

* Statistically significant values.

Aortic false lumen is a typical feature of AAAD (15). We noticed changes in coagulation function in AAAD patients, and some studies suggested that EOS might be involved in thrombosis (16, 17). Comparisons between EOS groups were significant for the five blood coagulation items. Patients with 0.00 × 10^9^/L EOS counts had higher PT [(12.8 s, IQR 11.8–14.35 s) vs. (12.4 s, IQR 11.5–13.9 s), *P* < 0.001], APTT [(30.4 s, IQR 26.9–39.3 s) vs. (29.3 s, IQR 26.8–34.55 s), *P* = 0.004], INR [(1.12 s, IQR 1.03–1.25 s) vs. (1.09 s, IQR 1.00–1.22 s), *P* < 0.001], TT [(19.3 s, IQR 17.5–22.1 s) vs. (18.6 s, IQR 16.9–20.9 s), *P* < 0.001], and lower fibrinogen levels [(2.1 g/L, IQR 1.6–2.7 g/L) vs. (2.4 g/L, IQR 1.7–3.3 g/L), *P* < 0.001] (Table 6). Furthermore, EOSs were observed in thrombi in the false lumen of the aorta (Supplementary Figure S2). Therefore, decreased peripheral blood EOS counts may be due to the involvement of EOSs in aortic false lumen thrombosis.

**Table 6:** Five blood coagulation items in both eosinophil groups.

Variables	Total (*n* = 1180)	EOS of 0.00 (*n* = 681)	EOS > 0.00 (*n* = 499)	*P* value
PT (10–15 s)	12.6 (11.7–14.2)	12.8 (11.8–14.35)	12.4 (11.5–13.9)	<0.001*
INR (0.8–1.3 s)	1.11 (1.02–1.24)	1.12 (1.03–1.25)	1.09 (1.00–1.22)	0.001*
TT (16–18 s)	18.9 (17.2–21.5)	19.3 (17.5–22.1)	18.6 (16.9–20.9)	0.001*
APTT (23–27 s)	29.9 (26.9–37)	30.4 (26.9–39.3)	29.3 (26.8–34.55)	0.004*
D-dimer (0–0.5 mg/L)	5.21 (2.83–11.74)	5.28 (3.04–11.44)	4.96 (2.43–12.07)	0.207
Platelet (100–400 × 10^9^/L)	137 (98.25–177)	125 (86–163)	157 (123–201)	<0.001*
Fibrinogen (2–4 g/L)	2.2 (1.6–3.0)	2.1 (1.6–2.7)	2.4 (1.7–3.3)	<0.001*

PT, prothrombin time; INR, international normalized ratio; TT, thrombin time; APTT, activated partial thromboplastin time.

* Statistically significant values.

## Discussion

Our study found that EOS counts in the peripheral blood of patients with AAAD were significantly lower; up to 81.1% patients EOS counts below 0.02 × 10^9^/L and 57.7% of patients had undetectable EOS counts (0.00 × 10^9^/L), concomitant with higher mortality and longer ICU treatment days. When we compared Siglec-8 expression in the control group with AAAD patients (preoperative EOS counts = “0.00”), and also histological examinations, we observed no evidence that EOSs accumulated in the aortic interstitial spaces of patients with AAAD. From statistical analyses of coagulation functions (APTT, TT, INR, PT, and Fibrinogen), D-dimer levels, and platelet counts in patients with AAAD, lower EOS counts tended to represent worse coagulation functions, higher D-dimer levels, and lower platelet counts. Furthermore, infiltrating EOSs were observed from the thrombus in the false lumen. Therefore, EOSs may be recruited from the peripheral blood into the false lumen and be associated with thrombus formation.

Aortic dissection is characterized by damage and remodeling of the aortic media leading to secondary thrombosis and inflammation, with systemic signs of inflammatory activation and local inflammatory cell infiltration (10, 18). Inflammatory cells, such as neutrophils (11), lymphocytes (19), and macrophages (20) have been investigated during AAAD pathogenesis, but EOS studies are lacking. EOSs are not only inflammatory effector cells, but they exert several immune regulatory functions. EOS counts in a large CVD patient cohort and experimental rat data on aortic aneurysms support the conclusion that EOS exert major protective roles in CVD (12). EOS deficiency increased abdominal aortic aneurysm growth, inflammatory cell lesion levels, angiogenesis, elastic rupture of the arterial wall, lesion cell apoptosis, SMC loss, and M1 macrophage marker expression (14). False lumen formation and subsequent thrombosis are important features in AAAD acute phases. Reports of thrombotic events in patients with EOS-related disease confirmed EOS involvement in thrombosis, i.e., promoting thrombosis through eosinophilic extracellular traps, thereby enhancing platelet activation, leading to atherosclerosis and stable thrombosis (16). Additionally, EOS may be involved in coronary thrombosis in acute myocardial infarction *via* inflammatory mechanisms (21). Activated PLT is associated with EOS pathology in several diseases, including asthma and hyper-eosinophilic syndrome (22). PLT is activated by EOS particles, major basic protein, and EOS peroxidase (23). Our data also suggested that EOS may be involved in false lumen thrombus formation in patients with AAAD.

Whether EOSs promote vascular injury, induce pro-inflammatory effects, or are simply recruited to tissue injury sites remain unclear. The main goal of AAAD surgery is to prevent fatal complications, and the more severe the preoperative vascular injury, the more likely it is to cause rupture, cardiac tamponade, and poor perfusion. Additionally, damaged vessels also make graft anastomosis more difficult during surgical treatment, resulting in postoperative complications such as bleeding and infection. Decreased EOS percentages may indicate severe vascular injury and more extensive thrombosis, therefore, EOS counts may be useful in assessing the extent of preoperative aortic injury in patients with AAAD.

### Study limitations

Our research had some limitations. Most AAAD patients were first diagnosed in local hospitals, therefore it was difficult to obtain accurate information on false lumen thrombosis. Similarly, information on the effects of false lumen thrombosis on postoperative in-hospital survival rates are lacking. Partial thrombosis of the false cavity is an important independent predictor of mortality in patients with type B dissection, but it does not affect the long-term survival rate of AAAD survivors after discharge. Blood flow and thrombus may coexist, and most patients had EOS counts below the lower limit of detection, therefore it was difficult to quantify associations between false lumen thrombosis and the degree of EOS reduction through limited specimen. Additionally, in considering intraoperative bleeding, blood transfusion, and postoperative anti-infection treatment, we did not continuously monitor EOS counts during hospitalization. Therefore, when EOSs leave the peripheral blood system, are they deposited in a thrombus or elsewhere, or are they destroyed or degraded? Furthermore, peripheral blood eosinophil counts have a circadian rhythm (peaked during nighttime) (24), the time of drawing blood may affect results. These questions require further investigation.

## Conclusions

Peripheral blood EOS counts may be valid indicators for preoperative risk assessment in patients with AAAD. Circulating EOS levels below detection limits may not only indicate thrombosis, but may be significant in predicting AAAD severity and prognosis in patients with AAAD. Therefore, a rapid, simple, and low-cost peripheral blood EOS count test can be used to effectively assess preoperative risk in patients with AAAD.

## Data Availability

The original contributions presented in the study are included in the article/Supplementary Material, further inquiries can be directed to the corresponding author/s.
