# Supplementary material for: The role of peripheral blood eosinophil counts in acute Stanford type A aortic dissection patients
Source: Front Surg. 2022 Aug 30;9:969995. doi: 10.3389/fsurg.2022.969995 (PMC9468214; doi:10.3389/fsurg.2022.969995)
Supplement: Supplementary file 1 [file Data_Sheet_1_v1.pdf]

Supplementary Materials and Methods

Western blotting and histological assessment

We used fresh AAAD samples from six patients (three males and three females) and normal ascending aortas from six patients underwent coronary artery bypass grafting (three males and three females, no aortic wall calcification or lesions were found during preoperative and intraoperative examination). All AAAD patients preoperative peripheral blood EOS counts were “0.00”, whereas EOS counts for the control group were $0.02–0.52\times10^9$/L. Half of the sample was used to prepare tissue homogenates for western blotting and the remainder for histology analysis. Samples were immersed in 4% paraformaldehyde for 24 h, dehydrated, embedded in paraffin, sectioned into 4 μm sections, and stained in hematoxylin & eosin (H&E).

Tissue proteins were separated using sodium dodecyl sulfate-polyacrylamide gel electrophoresis and transferred to nitrocellulose membranes. Membranes were blocked in 5% non-fat milk for 2 h at room temperature, then incubated overnight at 4°C with primary antibodies against Siglec-8 or GAPDH. After washing, membranes were incubated for 1–2 h at room temperature with fluorescently labeled anti-rabbit or anti-mouse secondary antibodies. Blots were imaged using an Odyssey infrared imaging system (Li-Cor). Densitometry was performed using ImageJ software. Siglec-8 expression levels were expressed as the ratio of Siglec-8/β-tubulin.

Supplementary Figures

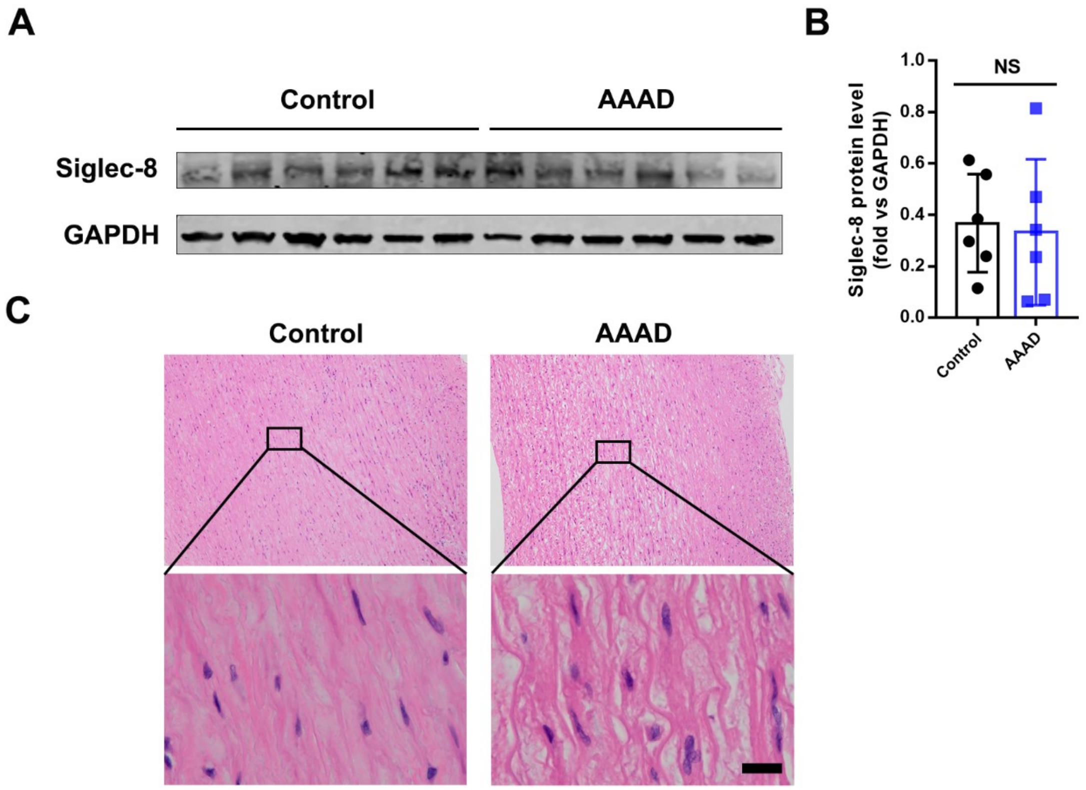

Figure 1. Eosinophil investigations in aortic samples. (A) Western blotting was used to detect Siglec-8 expression. (B) Quantitative assessment of Siglec-8 expression levels. NS = no significant difference. (C) Representative hematoxylin & eosin stained cross-section images of the ascending aorta from the control group and patients with AAAD. Scale bar = 20 μm.

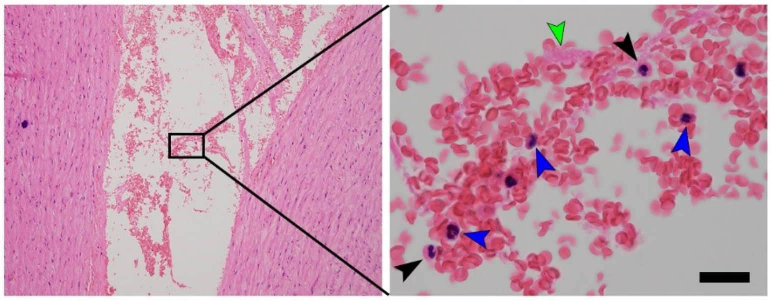

Figure 2. Eosinophils are involved in thrombosis. Representative hematoxylin & eosin stained cross-section images of the ascending aorta false lumen. The green arrow indicates thrombi, black arrows indicate neutrophils, and blue arrows indicate eosinophils. The cytoplasm in neutrophils is clear but is dark and diffuse in eosinophils. Scale bar = 20 μm.
